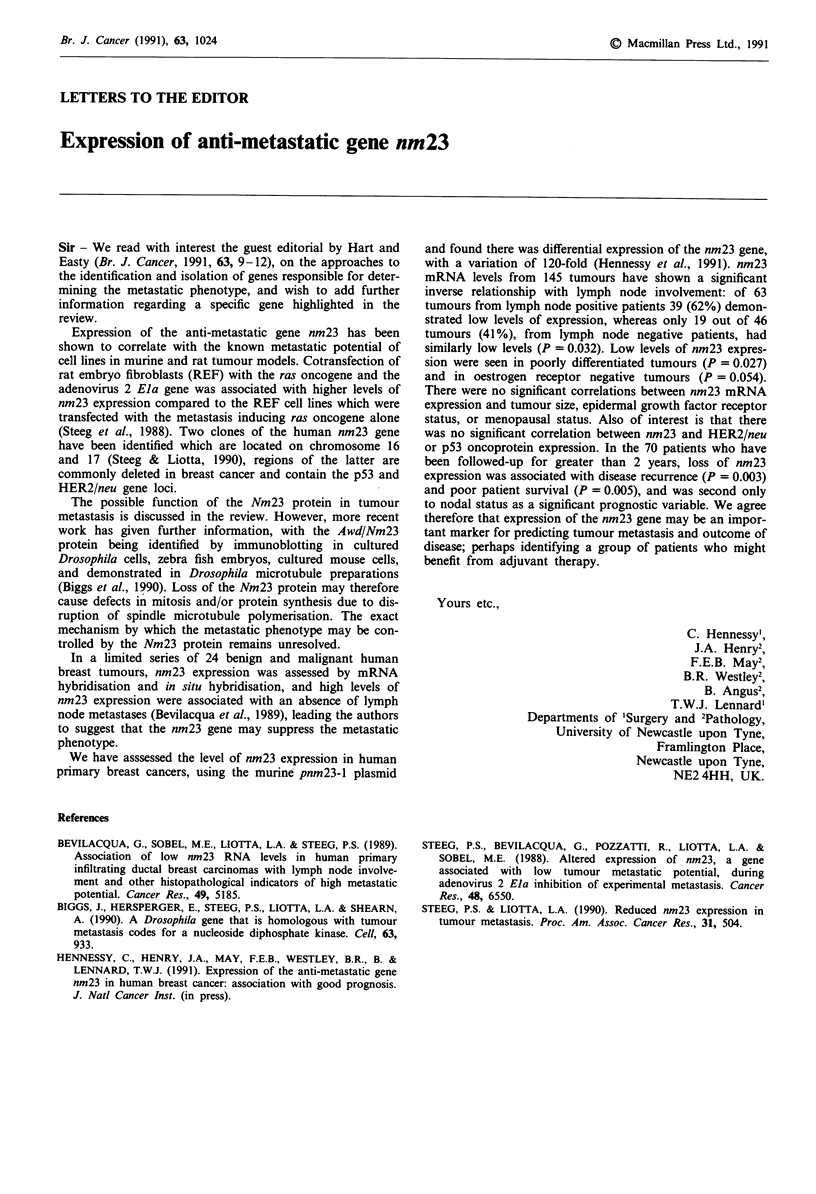# Expression of anti-metastatic gene nm23.

**DOI:** 10.1038/bjc.1991.223

**Published:** 1991-06

**Authors:** C. Hennessy, J. A. Henry, F. E. May, B. R. Westley, B. Angus, T. W. Lennard


Br. J. Cancer (1991), 63, 1024 © Macmillan Press Ltd., 1991

### LETTERS TO THE EDITOR

### Expression of anti-metastatic gene *nm*23

Sir – We read with interest the guest editorial by Hart and Easty (*Br. J. Cancer*, 1991, **63**, 9–12), on the approaches to the identification and isolation of genes responsible for determining the metastatic phenotype, and wish to add further information regarding a specific gene highlighted in the review.

Expression of the anti-metastatic gene *nm*23 has been shown to correlate with the known metastatic potential of cell lines in murine and rat tumour models. Cotransfection of rat embryo fibroblasts (REF) with the *ras* oncogene and the adenovirus 2 *Ela* gene was associated with higher levels of *nm*23 expression compared to the REF cell lines which were transfected with the metastasis inducing *ras* oncogene alone (Steeg *et al.*, 1988). Two clones of the human *nm*23 gene have been identified which are located on chromosome 16 and 17 (Steeg & Liotta, 1990), regions of the latter are commonly deleted in breast cancer and contain the p53 and HER2/*neu* gene loci.

The possible function of the *Nm*23 protein in tumour metastasis is discussed in the review. However, more recent work has given further information, with the *Awd/Nm*23 protein being identified by immunoblotting in cultured *Drosophila* cells, zebra fish embryos, cultured mouse cells, and demonstrated in *Drosophila* microtubule preparations (Biggs *et al.*, 1990). Loss of the *Nm*23 protein may therefore cause defects in mitosis and/or protein synthesis due to disruption of spindle microtubule polymerisation. The exact mechanism by which the metastatic phenotype may be controlled by the *Nm*23 protein remains unresolved.

In a limited series of 24 benign and malignant human breast tumours, *nm*23 expression was assessed by mRNA hybridisation and *in situ* hybridisation, and high levels of *nm*23 expression were associated with an absence of lymph node metastases (Bevilacqua *et al.*, 1989), leading the authors to suggest that the *nm*23 gene may suppress the metastatic phenotype.

We have asssessed the level of *nm*23 expression in human primary breast cancers, using the murine *pnm*23-1 plasmid and found there was differential expression of the *nm*23 gene, with a variation of 120-fold (Hennessy *et al.*, 1991). *nm*23 mRNA levels from 145 tumours have shown a significant inverse relationship with lymph node involvement: of 63 tumours from lymph node positive patients 39 (62%) demonstrated low levels of expression, whereas only 19 out of 46 tumours (41%), from lymph node negative patients, had similarly low levels ($P = 0.032$). Low levels of *nm*23 expression were seen in poorly differentiated tumours ($P = 0.027$) and in oestrogen receptor negative tumours ($P = 0.054$). There were no significant correlations between *nm*23 mRNA expression and tumour size, epidermal growth factor receptor status, or menopausal status. Also of interest is that there was no significant correlation between *nm*23 and HER2/*neu* or p53 oncoprotein expression. In the 70 patients who have been followed-up for greater than 2 years, loss of *nm*23 expression was associated with disease recurrence ($P = 0.003$) and poor patient survival ($P = 0.005$), and was second only to nodal status as a significant prognostic variable. We agree therefore that expression of the *nm*23 gene may be an important marker for predicting tumour metastasis and outcome of disease; perhaps identifying a group of patients who might benefit from adjuvant therapy.

Yours etc.,

C. Hennessy[1],
J.A. Henry[2],
F.E.B. May[2],
B.R. Westley[2],
B. Angus[2],
T.W.J. Lennard[1]
Departments of [1]Surgery and [2]Pathology,
University of Newcastle upon Tyne,
Framlington Place,
Newcastle upon Tyne,
NE2 4HH, UK.

**References**

BEVILACQUA, G., SOBEL, M.E., LIOTTA, L.A. & STEEG, P.S. (1989). Association of low *nm*23 RNA levels in human primary infiltrating ductal breast carcinomas with lymph node involvement and other histopathological indicators of high metastatic potential. *Cancer Res.*, **49**, 5185.

BIGGS, J., HERSPERGER, E., STEEG, P.S., LIOTTA, L.A. & SHEARN, A. (1990). A *Drosophila* gene that is homologous with tumour metastasis codes for a nucleoside diphosphate kinase. *Cell*, **63**, 933.

HENNESSY, C., HENRY, J.A., MAY, F.E.B., WESTLEY, B.R., B. & LENNARD, T.W.J. (1991). Expression of the anti-metastatic gene *nm*23 in human breast cancer: association with good prognosis. *J. Natl Cancer Inst.* (in press).

STEEG, P.S., BEVILACQUA, G., POZZATTI, R., LIOTTA, L.A. & SOBEL, M.E. (1988). Altered expression of *nm*23, a gene associated with low tumour metastatic potential, during adenovirus 2 *Ela* inhibition of experimental metastasis. *Cancer Res.*, **48**, 6550.

STEEG, P.S. & LIOTTA, L.A. (1990). Reduced *nm*23 expression in tumour metastasis. *Proc. Am. Assoc. Cancer Res.*, **31**, 504.